# Two novel genes *TOX3* and *COL21A1* in large extended Malay families with nonsyndromic cleft lip and/or palate

**DOI:** 10.1002/mgg3.635

**Published:** 2019-03-28

**Authors:** Nurul Syazana Mohamad Shah, Sarina Sulong, Wan Azman Wan Sulaiman, Ahmad Sukari Halim

**Affiliations:** ^1^ Reconstructive Science Unit, School of Medical Sciences Universiti Sains Malaysia Kubang Kerian Kelantan Malaysia; ^2^ Human Genome Centre, School of Medical Sciences Universiti Sains Malaysia Kubang Kerian Kelantan Malaysia; ^3^ Hospital Universiti Sains Malaysia Kubang Kerian Kelantan Malaysia

**Keywords:** cleft lip and/or palate, copy number, genetic, linkage, microarray

## Abstract

**Background:**

Nonsyndromic cleft lip and/or palate is one of the most common human birth defects worldwide that affects the lip and/or palate. The incidence of clefts varies among populations through ethnic, race, or geographical differences. The focus on Malay nonsyndromic cleft lip and/or palate (NSCL/P) is because of a scarce report on genetic study in relation to this deformity in Malaysia. We are interested to discuss about the genes that are susceptible to cause orofacial cleft formation in the family.

**Methods:**

Genome‐wide linkage analysis was carried out on eight large extended families of NSCL/P with the total of 91 individuals among Malay population using microarray platform. Based on linkage analyses findings, copy number variation (CNV) of *LPHN2*, *SATB2*, *PVRL3*, *COL21A1,* and *TOX3* were identified in four large extended families that showed linkage evidence using quantitative polymerase chain reaction (qPCR) as for a validation purpose. Copy number calculated (CNC) for each genes were determined with Applied Biosystems CopyCallerTM Software v2.0. Normal CNC of the target sequence expected was set at two.

**Results:**

Genome‐wide linkage analysis had discovered several genes including *TOX3* and *COL21A1* in four different loci 4p15.2‐p16.1, 6p11.2‐p12.3, 14q13‐q21, and 16q12.1. There was significant decreased, *p* < 0.05 of *SATB2*, *COL21A1, *and *TOX3* copy number in extended families compared to the normal controls.

**Conclusion:**

Novel linkage evidence and significant low copy number of *COL21A1 *and *TOX3 *in NSCLP family was confirmed. These genes increased the risks toward NSCLP formation in that family traits.

## 1 INTRODUCTION

Nonsyndromic cleft lip with or without palate (NSCLP) is a malformation without any other signs or symptoms of atypical condition such as abnormal physical appearance or psychological disease (Wallace, Arellano, & Gruner, 2011). Cleft lip and/or palate are one of the most common human embryonic disorder reported in Western countries and the second most common birth deformities among the babies (Muhamad & Azzaldeen, 2012; Shaw, Croen, & Curry, 1991). It arises in about one per 500 to 1,000 live births through ethnic and geographic differences (Muhamad & Azzaldeen, 2012; Murray, 2002). Birth prevalence and incidence of orofacial clefts vary among populations based on ethnic background (Cooper, Ratay, & Marazita, 2006).

Linkage analysis of multiplex families and association studies using either case–control or family‐based designs have become the primary methods for identifying potential genes for CL/P (Zeiger et al., 2003). Linkage studies have successfully revealed several different candidate loci studied in different populations. For example, a consanguineous family with 17 members was reported to have a homozygous 237‐kb deletion at locus 1p31 among the family members that cause cleft lip (Yıldırım, Kerem, Köroğlu, & Tolun, 2014). There are a lot of studies that revealed the causative genes to nonsyndromic cleft lip palate (NSCLP) formation such as *TGF *family, *FGF *family, cleft lip, and palate have been associated transmembrane protein 1 (*CLPTM1*), special AT‐rich sequence‐binding protein 2 (*SATB2*), and small ubiquitin‐like modifier 1 (*SUMO1*) and *IRF6 *(Carter et al., 2010; Nie, Luukko, & Kettunen, 2006; Pauws & Stanier, 2007; Scapoli et al., 2005).

Furthermore, multiple genome‐wide association study (GWAS) and relative extension studies had identified 22 susceptible loci in NSCLP including *IRF6* at 1q32 locus (Beaty et al., 2013; Leslie et al., 2016; Ludwig et al., 2016; Yu et al., 2017). Recent genomic study carried out among the cleft palate (CPO) of African population found two novel loci on chromosome 2 near *CTNNA2* and on chromosome 19 in *SULT2A1* (Butali et al., 2018). In addition, the emergence of next generation sequencing accelerates the finding of new loci associated with orofacial cleft (Cai et al., 2017). Copy number changes (CNC) analysis in exome sequencing has identified two CNC in *ADH7* and *AHR* in two multiplex families from Honduran population, whereas these two genes play role in craniofacial development (Cai et al., 2017).

To date, there is scarce report on genetic study of NSCLP in Malaysia, particularly on the Malay race as a majority of Malaysian population. Previously, some common genes such as *MSX1*, *IRF6, *and *TGF‐β *have been reported to be associated with NSCLP formation but many more uncommon genes could be elucidated in this study. In addition, identification of specific mutation and causal genes could be a major contribution in understanding the pathogenesis of orofacial clefts and might aid in developing preventive strategies. Plus, these identifications would be helpful in screening high risk individuals for genetic counseling purpose.

## 2 MATERIALS AND METHODS

### 2.1 Subjects

Eight large extended Malay families with three–four generations consists of 91 individuals were included. Healthy individuals with no cleft lip and/or palate phenotype, no family history of clefts or syndromic diseases were included and acts as a normal control. All the blood withdrawal was undertaken during the home visit or appoinment at clinic. They have been referred to the craniofacial specialist before the blood was taken. This study was approved by the Research Ethics Committee (Human) of the Universiti Sains Malaysia, Health Campus, Malaysia; Reference No.: USMKK/PPP/JEPeM [258.3.(3)] and written informed consent was obtained from the participants or their parent/guardian.

### 2.2 Microarray analysis

DNA was extracted from 200 μl of heparinized peripheral blood for microarray analysis using Illumina Infinium HumanLinkage‐24 Beadchip. All the DNA samples were arrayed at St George's Hospital, University of London, using the Illumina technology.

### 2.3 Validation with Copy Number Variation (CNV) assay using quantitative PCR (QPCR)

#### 2.3.1 Gene selection

Four large extended families that showed suggestive or significant linkage evidence were selected for the validation step. Commercialized human DNA control was used as a calibrator, healthy individuals as normal controls (C1‐C6), and targeted samples including both the affected and unaffected family members.

### 2.4 Taqman® copy number assay selection

The SNPs that showed suggestive or significant linkage were identified using National Center for Biotechnology Information (NCBI) database. Linkage interval for chromosome position or target information for the selected SNPs were entered into Assay Search Tool – Single Tube TaqMan® Assays from Life Technologies website (https://www.lifetechnologies.com/) to obtain specific primer‐probe pair. Five selected sequences were *Homo sapiens *SATB2 NCBI location: Chr.2:200134223–200335989; cytoband: 2q33.1d [Hs07541174_cn], TOX3 NCBI location: Chr.16:52503999; cytoband: 16q12.1 [Hs03924205_cn], COL21A1 NCBI location: Chr.6:56247500; cytoband: 6p12.1 [Hs00783345_cn], LPHN2 NCBI location: Chr.1:81800443; cytoband: 1p31.1 [Hs00381445_cn], and PVRL3 NCBI location: Chr.3:104871745; cytoband: 3q13.11 [Hs03228815_cn].

#### 2.4.1 Quantitative PCR (qPCR)

qPCR was performed using TaqMan® Genotyping Master Mix for absolute quantitation of copy number for Latrophilin 2 (*LPHN2*; OMIM: 607018)*, SATB2 *(OMIM: 608148), Poliovirus Receptor‐Related 3 (*PVRL3*; OMIM: 607147), alpha chain of type XX1 collagen (*COL21A1*; OMIM: 610002), and TOX High Mobility Group Box Family Member 3 (*TOX3*; OMIM: 611416). RNase P as an endogenous control and negative control (NTC). NTC is a reaction mixture without DNA template used as a negative control. qPCR was run using ABI 7,500 system.

#### 2.4.2 CNV analysis

Copy number calculated (CNC) for the genes were determined with Applied Biosystems CopyCaller™ Software v2.0. Number of copies of the target sequence expected in the majority of samples was set at two. Samples were reviewed to achieve optimal experimental conditions where samples are of high quality, copy number, and reference assays have amplified and sample replicates have similar CT and ΔCT values. Accepted copy number calls should have confidence value > 95% and Z‐score < 1.75.

### 2.5 Bioinformatics analysis

#### 2.5.1 Linkage analysis

Linkage analyses were carried out using Easy Linkage Plus v5.08 software (Hoffmann & Lindner, 2005). It allows multipoint simulation studies, detection of Mendelian/non‐Mendelian genotyping errors, and Hardy–Weinberg equilibrium (HWE). Due to limited pedigree capabilities of linkage programs supported by Easy Linkage software and limited pedigree plots are available from Gene Hunter software, Easy Linkage Plus extends the Gene Hunter plots by showing marker names and their genetic position (Hoffmann & Lindner, 2005). Gene Hunter‐Multipoint Linkage Analysis v2.1r5 was used to do multipoint parametric and nonparametric analysis under both dominant and recessive mode of inheritance as previously described by Shah, Salahshourifar, Sulong, Wan Sulaiman, and Halim (2016).

### 2.6 Statistical analysis

Statistical analysis of data was performed using Mann–Whitney test with SPSS Statistics (SPSS 22, SPSS, Inc., USA). Statistical significance was determined for *p* value <0.05 and all data were expressed as mean ± standard deviation (SD).

## 3 RESULTS

### 3.1 Linkage analysis results

Two human genetic maps markers were used; Illumina 6K Linkage 24 deCODE and AFFY 100k Marshfield Human Sex‐Averaged for comparison purposes. All markers were in Hardy–Weinberg Equlibrium (HWE) with *p* value <0.05 and the overall call rate was >98% for all samples. Both nonparametric and parametric analyses were carried out and came out with NPL and LOD score respectively. From eight extended families tested, there were four families showed positive linkage either suggestive or significant linkage at different loci. Significant linkage was only detected in Family 100 with NPL score ≥ 3.6 meanwhile other families had suggestive linkage with NPL score between 2.2 and 3.5. However, LOD score was only detected in two families; Family 100 and Family 99 with suggestive linkage (Table 1).

**Table 1:** Protein coding genes identified in four families that showed linkage of evidence after microarray analysis. Genes highlighted in bold were selected for a validation

Family	Linkage interval	SNP	NPL	LOD	Genes of interest in linkage interval
50	1p31.1–p34.2	rs697590	2.40	–	*PTPRF, AKR1AI, SSBP3, NFIA, LPHN2*
	2q34–q36.3	rs1851328	2.49	–	*ERBB4, ABCA12, STK36, IHH, PAX3, SATB2*
58	6p11.2–p12.3	rs1925154	3.17	–	*REL, COL21A1, DST*
	16q12.1	rs1420533	2.56	–	*TOX3, RBL2, RPGRIP1L, FTO*
100	1p31.1–p31.3	rs400382	*3.64	2.16	*LPHN2, GFPT1*
	2q31.1–q35	rs1002207	*3.69	2.18	*ZNF533, DNAJC10, FSIP2, SATB2, SUMO1, SP3, HOXD4, HOXD3, HOXD2, MSTN, NRP2, ABCA12, ERBB4*
99	3q13.3–q13.33	rs1398748	–	2.03	*COX17, GSK3B, FSTL1, HEG1, ZNF, CD200, BOC, CBLB, BBX, IFT57, PVRL3*

*Significant NPL score detected in Family 100 at two loci. *Sequences: *SATB2* NCBI location: Chr.2:200134223–200335989; cytoband: 2q33.1d; *TOX3 *NCBI location: Chr.16:52503999; cytoband: 16q12.1; *COL21A1* NCBI location: Chr.6:56247500; cytoband: 6p12.1; *LPHN2* NCBI location: Chr.1:81800443; cytoband: 1p31.1 and *PVRL3* NCBI location: Chr.3:104871745; cytoband: 3q13.11.

All the SNPs that lay within the suggestive and significant linkage intervals were employed to identify the genes of interest using GeneBank and GeneDistiller 2014 database. Table 1 showed several protein coding genes identified from the single nucleotide polymorphisms (SNPs) of nonparametric linkage (NPL) and logarithm of odds (LOD) score. Five selected candidate genes were chosen for validation; *LPHN2*, *SATB2*, *PVRL3*, *COL21A1,* and *TOX3 *based on literature review that is related to any craniofacial deformities or embryonic development.

### 3.2 Validation of *LPHN2* and *SATB2* copy number

**Figure 1:**
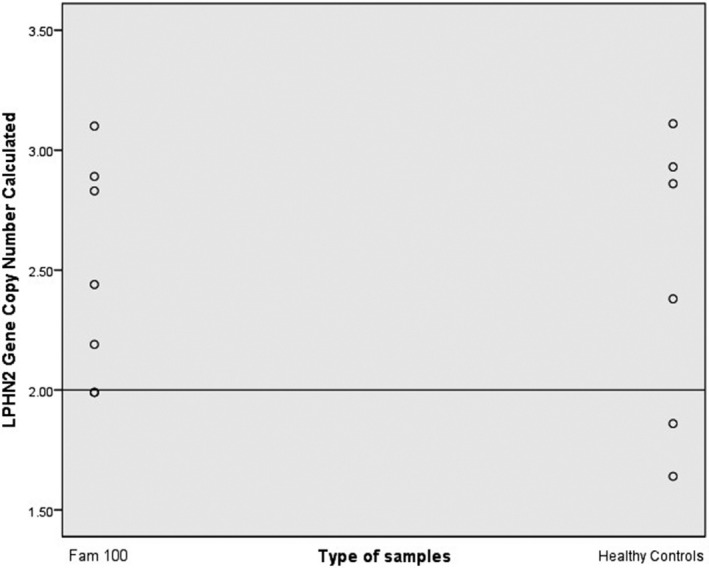
Scatter plot indicated *LPHN2* copy number value in Family 100 in comparison to the normal controls. The straight line depicted the calibrator set value at 2

Validation of *LPHN2 *by copy number variation (CNV) assay was done as a continuous finding of suggestive linkage found at 1p31 region in Family 100. From the findings, copy number calculated (CNC) for each individual in the Family 100 including affected and unaffected members showed CNC similar to control group, CNC > 2.50 or 2.49 ≥ CNC ≥1.50. Scatter plot data showed resemble distribution of CNC value between Family 100 and normal controls, that both having more than two copy numbers, depicted by a reference line at copy number of 2 (Figure 1). Statistical analysis had found no significant difference of copy number of *LPHN2* (*p* > 0.05) in Family 100 (2.49 ± 0.45) compared to the control group (2.46 ± 0.61), means that both groups had normal copy number for *LPHN2*. Therefore, *LPHN2* is regulated normally and not susceptible to NSCLP malformation.

**Figure 2:**
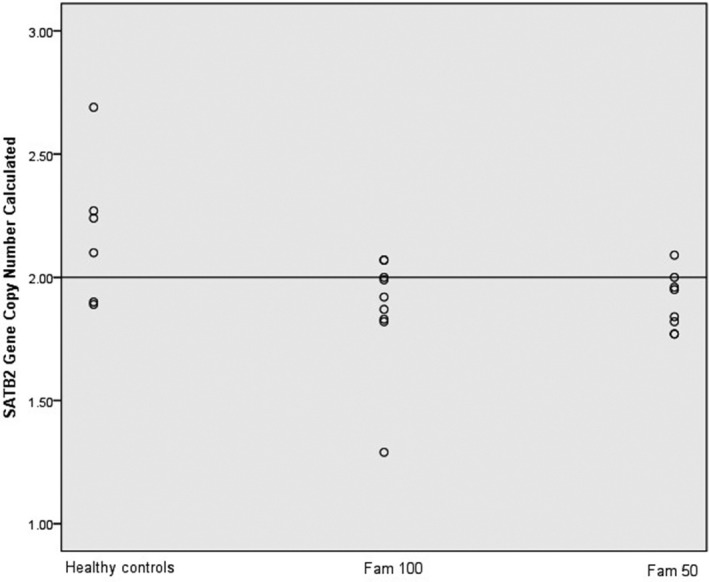
*SATB2* copy number in Family 50 and 100 in comparison to the healthy controls. The straight line depicted the calibrator set value at 2

Copy number of *SATB2* was determined for two families; Family 100 and Family 58. Scatter plot of the CNC data has shown distinct distribution of copy number between the affected NSCLP families from the normal control groups. Data plots were scattered below copy number of 2 in the affected families meanwhile normal group had higher than 2 copy numbers (Figure 2). There was a significant decreased in *SATB2* copy number in both Family 50 (1.90 ± 0.12) and Family 100 (1.87 ± 0.24), *p* < 0.05 compared to the normal controls. Therefore, this indicates *SATB2 *copy loss was confirmed among the families with strong family history of NSCLP cases.

### 3.3 Validation of *COL21A1* copy number

**Figure 3:**
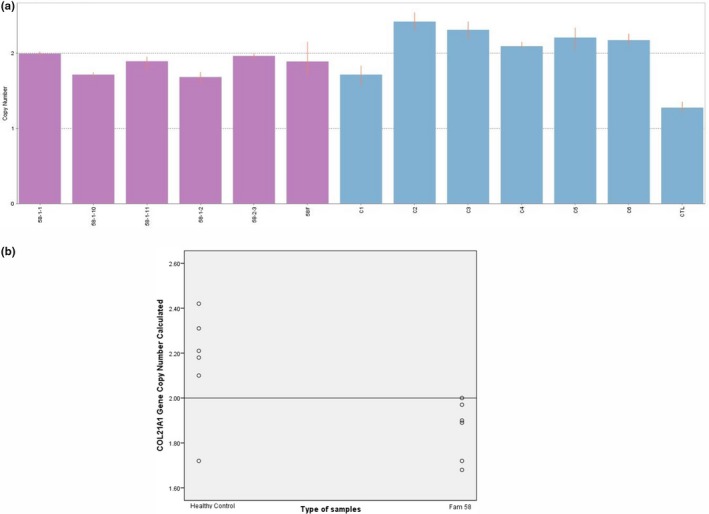
(a) Bars indicated copy number calculated (CNC) of *COL21A1 *in Family 58 (purple bars), normal control (blue bars), and calibrator (the first blue bar from right). (b) Scatter plot indicated copy number value of *COL21A1 *in Family 58 in comparison to the healthy controls. The straight line depicted the calibrator set value at 2

Copy number for *COL21A1 *was determined for Family 58 as it showed suggestive linkage in this family. Commercial human control that acts as calibrator had CNC < 1.50 (CNC = 1.28). Meanwhile, all the six healthy normal controls (C1‐C6) tested had copy number of 2.49 ≥ CNC ≥1.50. In total, all family members including two affected members (58‐F and 58–2‐3) had gene copy number within 2.49 ≥ CNC ≥1.50 (Figure 3). These findings indicated the *COL21A1 *copy number was similar in both normal controls and NSCLP family.

However, statistical analysis using scatter plot data had shown a distinct distribution of *COL21A1 *copy number between normal controls and Family 58. As depicted by the line at two copy numbers, gene copy number among the family members were lower than two compared to the normal controls that had copy number more than two (Figure 3). A significant decreased, *p* < 0.05 (*p* = 0.03) of *COL21A1 *copy number in Family 58 (1.86 ± 0.13) compared to the normal controls (2.16 ± 0.24) was confirmed.

### 3.4 Validation of *TOX3* copy number

**Figure 4:**
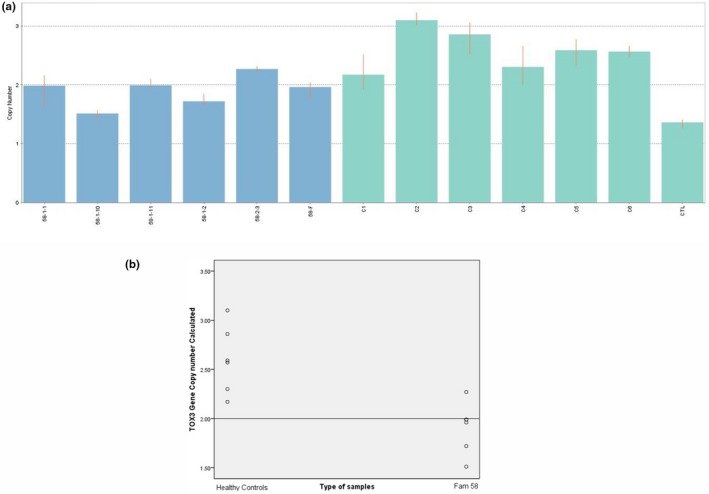
(a) Bars indicated copy number calculated (CNC) of *TOX3 *in Family 58 (blue bars), normal control (green bars), and calibrator (the first green bar from right). (b) Scatter plot indicated copy number value of *TOX3 *in Family 58 in comparison to the normal controls. The straight line depicted the calibrator set value at 2

*TOX3 *copy number was detected from Family 58 which had shown suggestive linkage on 16q12.1 region through genome‐wide linkage analysis. Commercial human control (calibrator) had CNC < 1.50 (CNC = 1.36) for *TOX3*. However, most of normal controls had CNC > 2.50. In Family 58, all family members including two affected members (58‐F and 58–2‐3) had 2.49 ≥ CNC ≥1.50 (Figure 4). None of them has reached the CNC > 2.50.

Scatter plot data showed a distinct distribution of *TOX3 *copy number between normal controls and Family 58, whereas low *TOX3* copy number was found in affected Family 58 (Figure 4). There was a significant decreased, *p* < 0.05 (*p* = 0.006) of *TOX3 *copy number in Family 58 (1.91 ± 0.26) compared to the normal controls (2.60 ± 0.34).

### 3.5 Validation of *PVRL3* copy number

**Figure 5:**
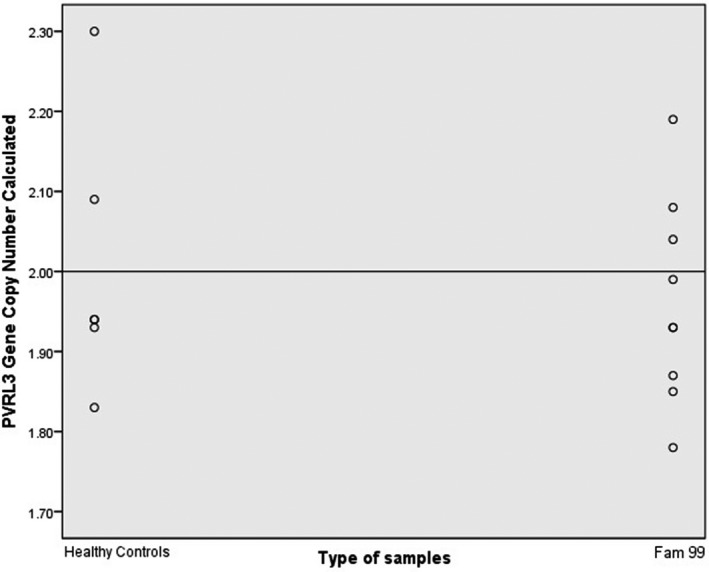
Scatter plot indicated copy number value of *PVRL3 *in Family 99 in comparison to the normal controls. The straight line depicted the calibrator set value at 2

Copy number of *PVRL3 *in Family 99 was predicted which had shown suggestive linkage on 3q13.3 region through genome‐wide linkage analysis. All the normal controls (C1–C6) and Family 99 expressed 2.49 ≥ CNC ≥1.50. Statistical analysis through scatter plot had found similar *PVRL3* copy number distribution between normal controls and Family 99 (Figure 5). Statistical significance had shown there was no significant difference, *p* > 0.05 (*p* = 0.55) of *PVRL3 *copy number in Family 99 (1.96 ± 0.13) compared to the normal controls (2.01 ± 0.17). This indicated that *PVRL3 *was not as a risk factor to the NSCLP formation in a family linkage analysis.

## 4 DISCUSSION

### 4.1 Nonsignificant copy number gain or loss in *LPHN2* and *PVRL3*

Despite linkage evidence has been attained through linkage analysis in Family 100, and detect *LPHN2 *as a target gene, copy number analysis did not show any positive finding on *LPHN2 *associated with clefting. The nonfunctional role of *LPHN2 *in NSCLP formation was confirmed since nonsignificant copy number gain or loss in the family has been detected in comparison to the normal control. The detection of linkage at 1p31 region for *LPHN2 *at the first level of microarray linkage analysis might reflect to the one individual in the family who had a high arch palate phenotype, but not the other NSCLP affected members. This is supported by the previous finding that has detected a deletion at 1p31 region that was associated with craniofacial abnormalities, particularly high‐arched palate (Beleza‐Meireles, Clayton‐Smith, & Tassabehji, 2014).

Linkage evidence at 3q13.3 region of NSCLP family identified candidate gene *PVRL3*. Early assumption that the selected NSCLP family had a lower copy number of *PVRL3 *compared to the normal individuals was not significantly proven. A normal detection of *PVRL3 *copy number in the NSCLP family reflected the finding by Sözen, Hecht, and Spritz (2008) that found no evidence of sequence variations in exons, intron, and noncoding sequences of *PVRL3 *among North American Caucasian population (Sözen et al., 2008). On contrary, other nectin‐family paralogues, *PVR*, *PVRL1, *and *PVRL2*, have been identified as candidate genes and play a role in etiology of NSCLP via genome‐wide linkage and association studies (Sözen et al., 2008; Warrington et al., 2006).

### 4.2 Copy number loss of known *SATB2*

As anticipated, absolute copy number loss of *SATB2 *in Family 50 and Family 100 was significantly attained in the family with affected members of nonsyndromic clefting compared to the normal individuals with no history of orofacial cleft. This finding plausibly explained the importance role of *SATB2 *in normal craniofacial development, which any loss of it may cause cleft defects. Similar to our finding, it has been found that *SATB2 *lay within the deleted region, with low copy number of *SATB2 *was detected in a patient with cleft compared to the both parents and control (Urquhart, Black, & Clayton‐Smith, 2009). Since very low significant copy number loss in NSCLP family compared to the normal individuals were detected, we expect that a microdeletion on 2q32‐q33 region might occur. However, microdeletion could not be identified with absolute qPCR due to the limitation in the power to determine DNA copy number (Sebat et al., 2004).

*SATB*2 is known to play a role in orofacial cleft particularly the cleft palate, involving the mutation at the chromosomal 2q32‐q33 region (FitzPatrick et al., 2003). Previous reports had identified the 2q32‐q33 region was critical for normal palatogenesis, whereas haploinsufficiency caused significant cleft palate defect (Brewer, Holloway, Zawalnyski, Schinzel, & FitzPatrick, 1998; 1999). *SATB2 *loss was reported to be associated with increased cell death at the developing jaw primordial including palate, therefore hindered regional development and finally caused craniofacial defects (Britanova et al., 2006). The loss of SATB2 gene copy number in the family of affected cleft indicated the risk and susceptibility of *SATB2 *to cause orofacial cleft in the family trait.

### 4.3 Novel finding on *COL21A1* copy number

The novel low copy number of 6p12.2 region in the affected family members has confirmed *COL21A1 *as one of the contributing genes to the NSCLP formation. This copy number finding has strengthened the linkage evidence detected previously via microarray. *COL21A1 *functions in maintaining the integrity of ECM. It has been reported that *COL21A1 *is a part and the smallest of FACIT family of collagen that is expressed in tissues expressing muscle phenotype such as skeletal muscle. It contains abundant ECM and enriched collagen I (Fitzgerald & Bateman, 2001). This co‐expression of collagen XXI and collagen I in tissues and muscles have important role in the organization of interstitial collagen fibrils by connecting it to other matrix components or cells (Chou, & Li, 2002; Fitzgerald, & Bateman, 2001). Therefore, detection of *COL21A1 *low copy number in NSCLP family possibly due to low concentration *COL21A1 *in DNA samples.

This condition might affect normal collagen activity in maintaining the tissue integrity of the lip and/or palate muscle and cause cleft lip deformity. As this gene has never been discussed or claimed to have a role in clefting, our finding is the first report of *COL21A1 *at 6p12.2 region that might be associated with nonsyndromic orofacial cleft. Similarly, one study on six Amish patients with multiplex human syndrome with cleft lip and palate anomaly has attained the candidate locus at a similar chromosome 6p12.2‐p12 but they harbored *ICK *missense mutation (Lahiry et al., 2009).

Since the evidence for linkage to this region was only detected in one of eight NSCLP families, this data could still provide an important significant output in a gene discovery to the occurrence of NSCLP, as it is known to be heterogeneous. Previously, two different studies has proposed that major clefting locus might be located at 6p and mutation in several genes such as *COL2A1*, *COL11A1, *and *COL11A2 *caused syndrome that was associated with cleft palate, Robin sequence, and micrognathia (Melkoniemi et al., 2003; Murray, 1995). On the other hand, it has also been found that the *COL2A1*, *COL11A1, *and *COL11A2 *were associated and influence the risk of nonsyndromic forms of cleft palate (Jugessur et al., 2009; Nikopensius et al., 2010). No one has reported the role of *COL21A1 *in association with nonsyndromic orofacial cleft so far but the mutation at the 6p region has been attained before.

Different gene copy number among individuals and populations reflects to the gene expression variation (Stranger et al., 2007). Many studies have reported that the change in gene copy number has led the cells modifying transcription process and change the expression level (Prestel, Feller, & Becker, 2010; Stranger et al., 2007). Hence, evidence for linkage between *COL21A1 *and potential cleft phenotype has been confirmed in this family. Loss of one copy of *COL21A1 *was clearly observed on the family members compared to the normal controls, so then we concluded that this *COL21A1 *might give a minor contribution to the NSCLP formation and could trigger the cleft occurrence in the family tree. This novel finding would help to shed light on the regulation of *COL21A1 *on the orofacial cleft formation.

### 4.4 Novel finding of low *TOX3* copy number

Lower *TOX3 *copy number at 16q12.1 has been achieved through CNV analysis in one affected family, Family 58. Currently, it has never been reported that the *TOX3 *was susceptible to nonsyndromic orofacial cleft occurrence. Therefore, this would be the first in which a finding revealed dysregulation of *TOX3 *in a family member of affected NSCLP, supporting the data of our microarray linkage analysis. Previous finding has found a deletion on similar locus of 16q12.1‐q13 on a patient of Pierre Robin sequence with median cleft palate (Schuffenhauer et al., 1992). Second, two different studies have also been reported to have mutation on 16q12.1‐q12.2 in syndromic Japanese patients with different phenotypes, and another one Germany patient with cleft of the soft palate anomaly, similarly *TOX3 *was one of the protein‐coding genes detected (Morisada et al., 2014; Shoukier et al., 2012).

Deletion on 16q12 region has been extensively studied in decades in relation to syndromic cases with various phenotypes and one of the craniofacial anomalies identified was high‐arched palate or cleft of the soft palate (Callen et al., 1993; Chang et al., 2010; Elder, Ferguson, & Lockhart, 1984; Shoukier et al., 2012). Therefore, there is high possibility that this mutation also occurred in nonsyndromic cleft cases but the specific mechanism of action of *TOX3 *confers craniofacial deformity risk was unclear. Through literatures, it has been discussed that the mutation of *TOX3 *was associated with breast cancer susceptibility (Jones et al., 2013; Udler et al., 2010). In addition, studies have been done in investigating the metastasis of breast cancer cells to bone.

The mechanism of *TOX3 *in triggering CL/P formation was unknown. But, it was likely that mutation of *TOX3 *interact with impaired *FGF*s in developing this deformity, which meant both of them deficit during the bone fusion in pre‐developmental stage. This was supported by our study on cellular level, whereas significant *FGF *down‐regulation was proven interfered normal embryonic craniofacial developmental process via FGF signaling (unpublished data). We hypothesized that reduced induction of *TOX3 *triggering FGF dysregulation in progression of lip and palate fusion and finally drive to the abnormal growth.

Validation through copy number analysis has strengthened the previous linkage evidence outcomes. Having significantly low copy number of these genes; *COL21A1 *and *TOX3 *in each selected NSCLP families compared to normal groups has increased the risks toward NSCLP formation in their family traits.

## CONFLICT OF INTEREST

The authors declare that there are no competing interests.
